# Banana (*Musa acuminata*) transcriptome profiling in response to rhizobacteria: *Bacillus amyloliquefaciens* Bs006 and *Pseudomonas fluorescens* Ps006

**DOI:** 10.1186/s12864-019-5763-5

**Published:** 2019-05-14

**Authors:** Rocío M. Gamez, Fernando Rodríguez, Newton Medeiros Vidal, Sandra Ramirez, Roberto Vera Alvarez, David Landsman, Leonardo Mariño-Ramírez

**Affiliations:** 1 Corporación Colombiana de Investigación Agropecuaria (AGROSAVIA), Centro de Investigación Tibaitatá, Km 14 Vía Mosquera, Bogotá, Colombia; 2 Universidad de la Sabana, Chía, Colombia; 3 National Center for Biotechnology Information, National Library of Medicine, National Institutes of Health, 8600 Rockville Pike, Bethesda, MD 20894-6075 USA

**Keywords:** Banana cv. Williams, *Musa acuminata*, Transcriptome, Genes, Plant growth promoting rhizobacteria (PGPR), *Bacillus amyloliquefaciens* Bs006, *Pseudomonas fluorescens* Ps006

## Abstract

**Background:**

Banana is one of the most important crops in tropical and sub-tropical regions. To meet the demands of international markets, banana plantations require high amounts of chemical fertilizers which translate into high farming costs and are hazardous to the environment when used excessively. Beneficial free-living soil bacteria that colonize the rhizosphere are known as plant growth-promoting rhizobacteria (PGPR). PGPR affect plant growth in direct or indirect ways and hold great promise for sustainable agriculture.

**Results:**

PGPR of the genera *Bacillus* and *Pseudomonas* in banana cv. Williams were evaluated. These plants were produced through in vitro culture and inoculated individually with two rhizobacteria, *Bacillus amyloliquefaciens* strain Bs006 and *Pseudomonas fluorescens* strain Ps006. Control plants without microbial inoculum were also evaluated. These plants were kept in a controlled climate growth room with conditions required to favor plant-microorganism interactions. These interactions were evaluated at 1-, 48- and 96-h using transcriptome sequencing after inoculation to establish differentially expressed genes (DEGs) in plants elicited by the interaction with the two rhizobacteria. Additionally, droplet digital PCR was performed at 1, 48, 96 h, and also at 15 and 30 days to validate the expression patterns of selected DEGs. The banana cv. Williams transcriptome reported differential expression in a large number of genes of which 22 were experimentally validated. Genes validated experimentally correspond to growth promotion and regulation of specific functions (flowering, photosynthesis, glucose catabolism and root growth) as well as plant defense genes. This study focused on the analysis of 18 genes involved in growth promotion, defense and response to biotic or abiotic stress.

**Conclusions:**

Differences in banana gene expression profiles in response to the rhizobacteria evaluated here (*Bacillus amyloliquefaciens* Bs006 and *Pseudomonas fluorescens* Ps006) are influenced by separate bacterial colonization processes and levels that stimulate distinct groups of genes at various points in time.

**Electronic supplementary material:**

The online version of this article (10.1186/s12864-019-5763-5) contains supplementary material, which is available to authorized users.

## Background

Banana (*Musa acuminata* Colla) belongs to the Monocotyledons class, Zingiberales order and to the Musaceae family, which is composed of the *Musa* and *Ensete* genera. The *Musa* genus consists of four sections or species groups: *Australimusa, Callimusa, Rhodochlamys* and *Eumusa* [1], although another section *Ingentimusa* has also been considered as part of *Musa* [2]. The species of the *Eumusa* section have the largest geographical dispersion, particularly *Musa acuminata* and *Musa balbisiana* [3]. Bananas are perennial crops that grow quickly and can be harvested throughout the year, being moreover, the largest monoculture worldwide. Commercial cultivation of banana requires large amounts of nitrogen and potassium followed by phosphorus, calcium and magnesium to maintain high yields [4, 5]. In 2014, bananas were planted around the world in an area covering approximately 5,393,811 ha. The average global production in 2014 was 114,130,151 tons per year, and yield was reported in 211,595 kg/ha. The largest banana-producing countries are India, Uganda, China, the Philippines and some Latin American countries. Ecuador is the largest producer in Latin America (5.1 million tons), followed by Brazil and Colombia [6].

Despite the widespread cultivation of bananas, little is known about the microbial rhizosphere of *Musa* spp. Recent studies have shown that microbial populations in banana can be influenced by the type of production system, e.g. agroforestry. Agroforestry increases the number of beneficial bacteria such as *Pseudomonas* spp. and *Stenotrophomonas* spp., and decreases infections caused by pathogens such as *Erwinia* spp. [7]. Organic fertilization based on *Bacillus amyloliquefaciens* NJN-6 has resulted in significant decreases in the incidence of *Fusarium oxysporum*, f.sp. cubense [8]. *Pseudomonas fluorescens* has been isolated from banana plantations infected with *Cylindrocladium* sp. and its suppression by *Pseudomonas fluorescens* has been demonstrated [9]. Additionally, various microorganisms including *Arthrobacter*, *Azospirillum*, *Bradyrhizobium*, *Bacillus*, *Pseudomonas*, *Azotobacter* and *Paenibacillus,* among others, are known plant-growth promoting rhizobacteria (PGPRs) [10, 11]. PGPRs include a wide range of bacteria that may colonize roots with the ability to enhance plant growth by increasing seed germination, plant weight and crop yield [12]. It has been shown that some PGPRs, when colonizing plant roots, can transform certain compounds exuded by roots into plant growth-promoting substances that manipulate plant hormone signaling pathways [10, 12–15]. Seed treatment with PGPRs leads to increased plant growth [16–18] and fruit production [19, 20]. *Bacillus* spp. and *Pseudomonas* spp. have been reported in the banana rhizosphere [8], and the presence of these PGPRs, has been associated with plant production of exudates that favors the colonization and formation of biofilms in banana roots [20]. Recently, and previous to this study, the genomes of two PGPRs, *Pseudomonas fluorescens* strain Ps006 and *Bacillus amyloliquefaciens* strain Bs006 associated with banana cv. Williams seedlings have been isolated and characterized [21, 22]. According to the aforementioned, the aim of this study is to explore the effect of these two rhizobacteria, *Pseudomonas fluorescens* Ps006 and *Bacillus amyloliquefaciens* Bs006, on the banana cv. Williams seedling transcriptome and contribute to the current understanding of the banana plant-microorganism interactions in relation to growth promotion.

## Results

### Effect of PGPR on banana growth

**Fig. 1:**
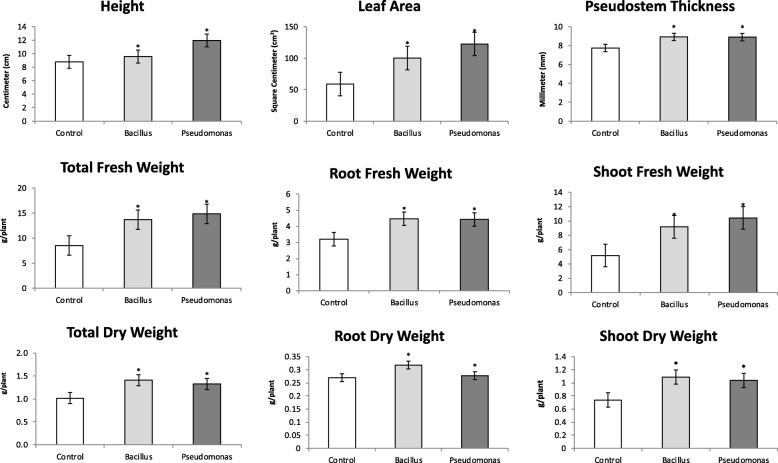
Biological assays. Effect of *Bacillus amyloliquefaciens* (Bs006) and *Pseudomonas fluorescens* (Ps006) on banana growth compared to the control (no PGPR inoculation). Nine variables were evaluated in 55-days-old seedlings after inoculation: height (cm), leaf area (cm^2^), pseudostem thickness (mm), total fresh weight, including shoot and root (g/plant), root fresh weight (g/plant), shoot fresh weight (g/plant), total dry weight (g/plant), and shoot dry weight (g/plant). Samples inoculated with *Bacillus amyloliquefaciens*, *Pseudomonas fluorescens*, and the control (no PGPR inoculation) are depicted as light-grey, dark-grey and white, respectively. Asterisk (*) denotes *p*-value < 0.0001 in treated samples compared to the control

The inoculation effect of *Bacillus amyloliquefaciens* strain Bs006 and *Pseudomonas fluorescens* strain Ps006 on banana plant growth was investigated 55 days post-inoculation at the greenhouse level. Eight variables were evaluated: height (cm), leaf area (cm^2^), pseudostem thickness (mm), total fresh weight (g/plant), fresh root weight (g/plant), fresh shoot weight (g/plant), total dry weight (g/plant), root dry weight (g/plant) and shoot dry weight (g/plant) (Fig. 1). All variables were significantly higher in samples inoculated with *B. amyloliquefaciens* or *P. fluorescens* compared to the control (no PGPR inoculation). These results agree with the beneficial effects of PGPR on plant development, quantity, quality and yield [23].

### Differential gene expression in response to PGPR

**Fig. 2:**
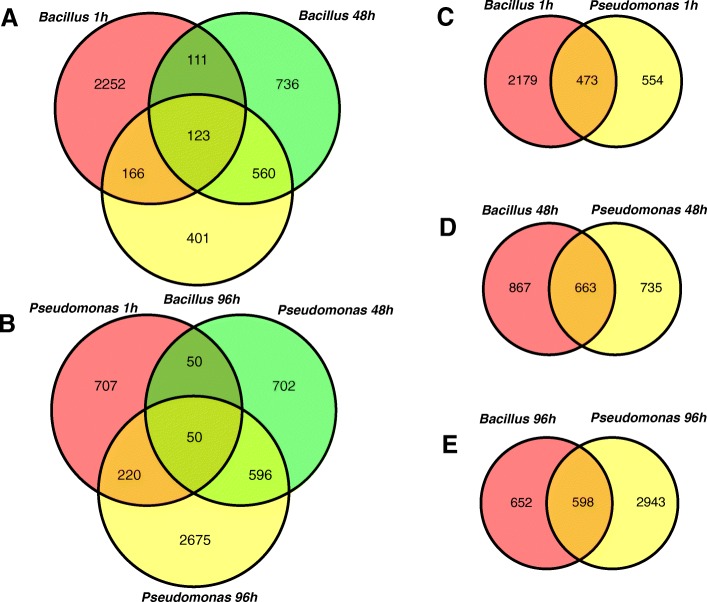
Venn diagram of differentially expressed genes (DEGs) at 1, 48 and 96 h post-inoculation with *Bacillus amyloliquefaciens* (Bs006) (**a**) and *Pseudomonas fluorescens* (Ps006) (**b**). Venn diagram of DEGs shared between *Bacillus amyloliquefaciens* and *Pseudomonas fluorescens* at 1 h (**c**), 48 h (**d**) and 96 h (**e**) post-inoculation

To better understand the direct and indirect interactions between banana roots and rhizobacteria, global transcriptome profiles were obtained at 1, 48 and 96 h post-inoculation with *B. amyloliquefaciens* Bs006 or *P. fluorescens* Ps006*.* Treated samples (3 biological replicates) were compared against the control (no PGPR inoculation) at 0 h post-inoculation, and genes were considered differentially expressed when the false discovery rate (FDR) was less than 0.05. The banana transcriptome response to *P. fluorescens* showed an increasing number of differentially expressed genes (DEGs) over time with 1027, 1398 and 3541 genes being changed at 1, 48, and 96 h, respectively. In contrast, the banana transcriptome response to *B. amyloliquefaciens* showed a decreasing number of DEGs over time with 2652, 1530 and 1250 genes being differentially expressed at 1, 48, and 96 h, respectively. (Additional files 1, 2, 3, 4: Figure S1,Table S2, S3, S4). These gene expression differences observed between the *Bacillus* and *Pseudomonas* treatments could also be influenced by the bacterial colonization level. To better understand this inverse temporal pattern between *Bacillus* and *Pseudomonas* on induced transcriptional responses, we identified genes differentially expressed in both treatments. The numbers of DEGs shared between treatments were 473, 663 and 598 at 1, 48 and 96 h post-inoculation, respectively (Fig. 2).

### Gene ontology enrichment analysis of DEGs

**Fig. 3:**
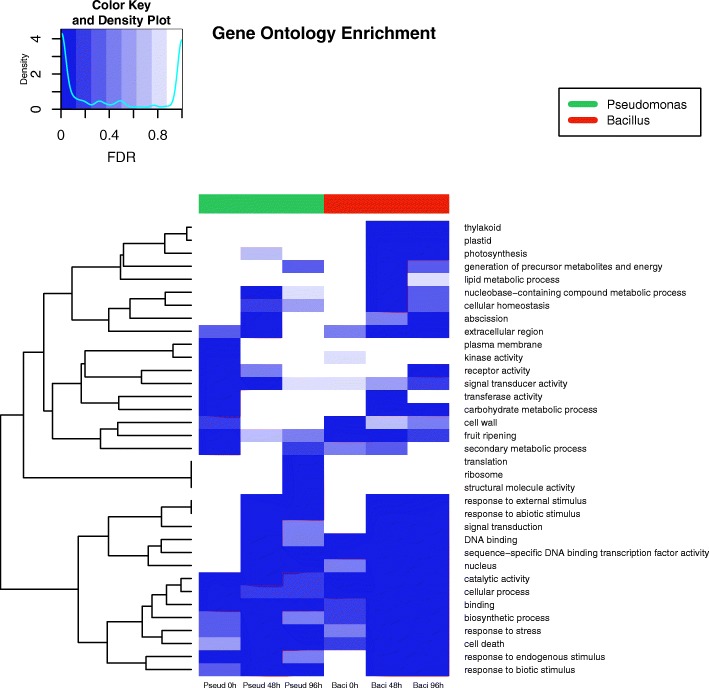
Hierarchical clustering and heat map of the gene ontology enrichment analysis results at 1, 48 and 96 h post-inoculation with *Bacillus amyloliquefaciens* (Bs006) and *Pseudomonas fluorescens* (Ps006)

A gene ontology (GO) enrichment analysis was performed across six treatments and these were compared with the reference control after the first hour to have a better understanding of the functional classes of DEGs for each treatment. Figure 3 shows the hierarchical clustering and significance of GO Plant Slim-terms over time for both PGPRs. *Bacillus* and *Pseudomonas* treatments at 1 h share similarities, meanwhile *Bacillus* at 48 and 96 h are also similar, suggesting differences in timing possibly due to levels of bacterial colonization or the induction of distinct metabolic pathways at different times triggered by the PGPR.

Gene ontology enriched terms can be grouped into four distinct classes: transcription factors (sequence-specific DNA binding transcription factor activity); genes involved in response to stimuli (stress, external, biotic, abiotic, endogenous); genes involved in cell proliferation (structural molecule activity, ribosome, translation); and genes involved in energy production (generation of precursor metabolites and energy, lipid metabolic process, plastid, thylakoid, photosynthesis) (Table 1). On the other hand, genes involved in cell proliferation are enriched only in *Pseudomonas* at 96 h, and genes involved in energy production are enriched only in *Bacillus* at 48 h. Transcription factors and genes involved in response to stimuli are enriched in samples inoculated with both rhizobacteria at multiple points in time: in *Pseudomonas* at 48 and 96 h, and in *Bacillus* at 48 and 96 h (Table 1).

**Table 1:**
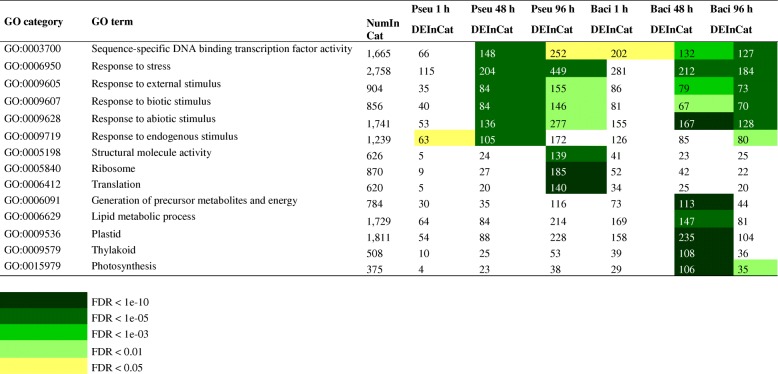
Gene ontology enrichment analysis. List of 14 gene ontology enriched terms with a FDR < 0.05 in at least one of the treatments: Pseu 1 h (*Pseudomonas fluorescens* 1 h post-inoculation), Pseu 48 h (*Pseudomonas fluorescens* 48 h post-inoculation), Pseu 96 h (*Pseudomonas fluorescens* 96 h post-inoculation), Baci 1 h (*Bacillus amyloliquefaciens* 1 h post-inoculation), Baci 48 h (*Bacillus amyloliquefaciens* 48 h post-inoculation), Baci 96 h (*Bacillus amyloliquefaciens* 96 h post-inoculation). NumInCat: number of expressed genes across all experiments (background) in each gene ontology category. DEInCat: number of differentially expressed genes for each gene ontology category in each treatment

### Transcription factors differentially expressed in response to PGPR

**Fig. 4:**
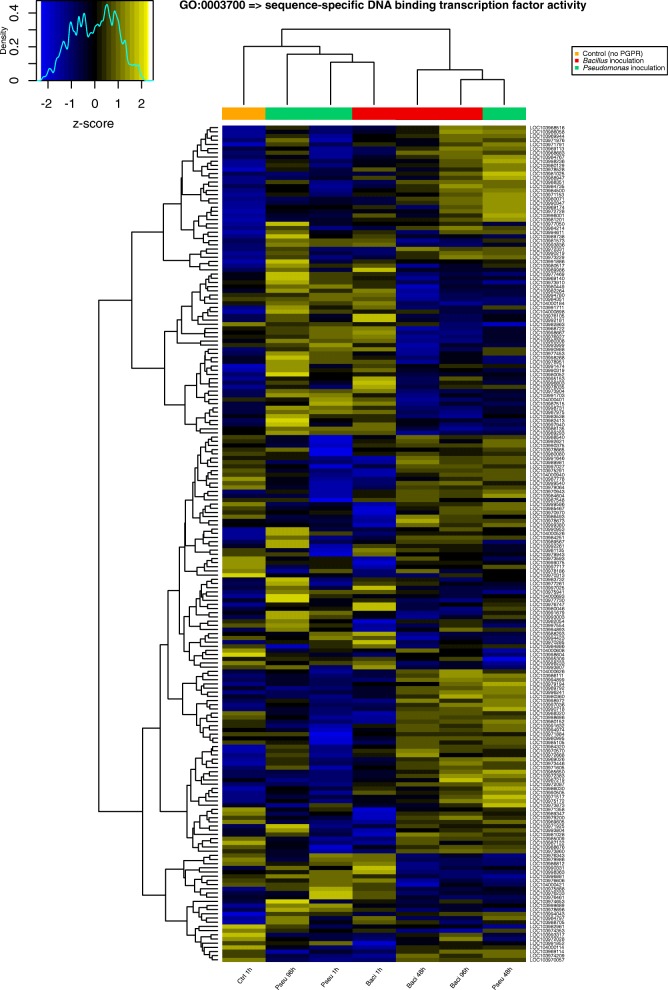
Hierarchical clustering and heat map of differentially expressed genes in the gene ontology category sequence-specific DNA binding transcription factor activity (GO:0003700). Samples inoculated with *Bacillus amyloliquefaciens* (Bs006), *Pseudomonas fluorescens* (Ps006), and the control (no PGPR inoculated) are depicted in red, green, and orange, respectively. Only top 200 genes (ranked by fold-change) are shown

Transcription factors (TFs) interact with specific DNA sequences to modulate gene expression levels. Additionally, a transcription factor is involved in developmental processes, response to intracellular signals, response to the environment, cell cycle control, and pathogenesis, among others. Differentially expressed transcription factors were found not to have large overlaps at the points in time evaluated. However, there was some overlap between strains Bs006 and Ps006 at similar points in time, but there were also a number of specific TFs elicited by each PGPR. It is clear that there are specific interactions and levels of response of TFs in the plant that vary with the PGPR *Bacillus amyloliquefaciens* (Bs006) or *Pseudomonas fluorescens* (Ps006), as well as with the times after inoculation. Interestingly, *Bacillus amyloliquefaciens* Bs006 starts with 171 differentially expressed TFs in the first hour post-inoculation. However, this effect tapers off in time, with only 30% of transcription factors differentially expresses after 96 h. In the case of *Pseudomonas fluorescens* Bs006, the effect is opposite. This treatment starts with 35 differentially expressed transcription factors one hour after inoculation, but these increase significantly to 182 genes at a later time after inoculation. This fact could also be closely related to the level of bacterial colonization over time. Figure 4 shows a hierarchical clustering and heat map of differentially expressed genes in the gene ontology category sequence-specific DNA binding transcription factor activity (GO:0003700).

### *Changes in banana gene expression in response to Bacillus amyloliquefaciens* Bs006 *and Pseudomonas fluorescens* Ps006 *evaluated using ddPCR*

**Fig. 5:**
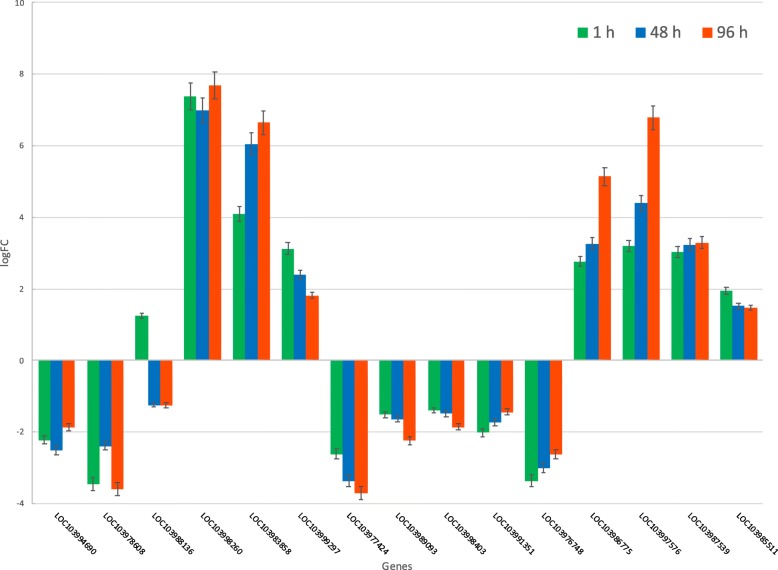
Gene expression trends for banana DEGs in response to *Bacillus amyloliquefaciens* (1 h, 48 h, 96 h). Gene expression trends for 15 experimentally validated banana genes that were differentially expressed with *Bacillus amyloliquefaciens* (Bs006) treatments at 1 h (green bars), 48 h (blue bars) and 96 h (red bars). The first 6 genes are common with plants treated with *Pseudomonas fluorescens*

**Fig. 6:**
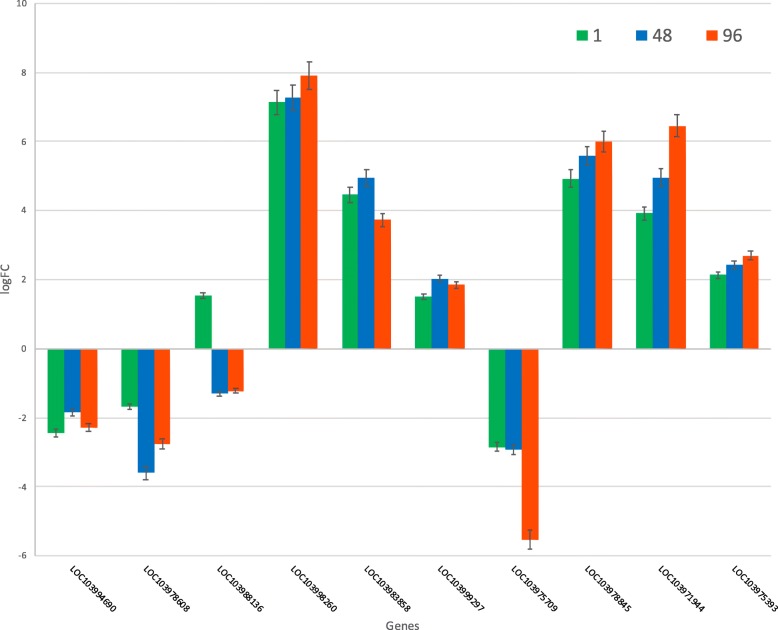
Gene expression trends for banana DEGs in response to *Pseudomonas fluorescens* (1 h, 48 h, 96 h). Gene expression trends for 10 experimentally validated banana genes that were differentially expressed with *Pseudomonas fluorescens* (Ps006) treatments at 1 h (green bars), 48 h (blue bars) and 96 h (red bars). The first 6 genes are common with plants treated with *Bacillus amyloliquefaciens*

The PGPRs *B. amyloliquefaciens* Bs006 and *P. fluorescens* Ps006 induced significant changes in the gene expression in banana cv. Williams seedlings. In this study experimental validation trials on 22 selected genes involved in plant growth promotion, defense and biotic or abiotic stresses were performed (Table 2). The expression levels of these 22 genes related to PGPR effects were validated using droplet digital PCR (ddPCR) and with this technique, it was confirmed that the genes evaluated are differentially expressed in the presence of the two rhizobacteria as shown also by RNA-seq. The experimentally validated genes were categorized into several types of functions associated with PGPR effects on growth: root growth, foliar growth, flowering, metabolism, defense, stress, photosynthesis and their trends were plotted (Figs. 5, 6). The RNA-Seq data showed that *Bacillus* induce a response earlier than *Pseudomonas* (Fig. 2). However, this difference in the timing of response between *Bacillus* and *Pseudomonas* was not observed by ddPCR. It is not clear why we were not able to observe a correlation between RNA-Seq and ddPCR

**Table 2:** Genes and sequences of primers used for validation by droplet digital PCR (ddPCR)

Locus	Gene name	Product length (nt)	Primer F (5′- > 3′ on plus strand)	Primer R (5′- > 3′ on minus strand)	Tm (Celsius)	G + C (%)
6 Genes: Six genes selected because they are differentially expressed in three temporary evaluation points (1 h, 48 h and 96 h) in *Bacillus* and *Pseudomonas* treatments						
LOC103994690	L-ascorbate oxidase homolog	139	TCTCACCACCAGCCAATTCC	ATGCAGCAGAGACACAACGA	60	50–55
LOC103978608	Wound-induced protein 1-like	70	CTAACATGGTCCTCGGCTCG	AGTACACTCAGATCGGCCCT	60	55–60
LOC103988136	Cytochrome C	94	AACATGTTGCCCTGCTTGTG	TCAGGCAAGAGCCCATGAAA	60	50
LOC103998260	Cytochrome P450 714B3-like	136	ACTCTTCTCACAAGGCCGAC	TCAACTCTGCCCATGACACA	60	50–55
LOC103983858	Ubiquinol oxidase 2, mitochondrial-like	115	GCGGCTTGTTTGGTGTTTCT	AGGAACGTACCCAATGCTGG	60	50–55
LOC103999297	Palmitoyl-acyl carrier protein thioesterase, chloroplastic	140	TTAAGGCGCAAGCACAAACC	CAGAAAGCTCCAGTCAGGCA	60	50–55
4 Genes: Four genes selected because they are differentially expressed in three temporary evaluation points (1 h, 48 h, 96 h) under *Pseudomonas* treatment						
LOC103971944	Caffeic acid 3-O-methyltransferase	144	GGTGGAGGAGACGCCATTTT	CGGAATCACGGGAAGCAAGT	60	55
LOC103975393	Heat shock cognate 70KDa protein	143	ACGAGGGTGAGAGGACAAGA	GGCGGACACGTTTATGATGC	60	55
LOC103975709	Small heat shock protein	100	GCCTCAGCGACTACCTTACC	ATGCTACTGTCGAAGGCTGG	60	55–60
LOC103978845	Truncated transcription factor CAULIFLOWER	108	GCTCTACGAGTACTCCACCG	TCCTGGGATTCAGTGTCTGC	60	55–60
12 Genes: Twelve genes selected because they are differentially expressed in three temporary evaluation points (1 h, 48 h, 96 h) under *Bacillus* treatment						
LOC103998403	Heat shock cognate 70KDa protein 8	71	ATCGGCCTAGGTGAAGATGG	TGTCGTCACCGGTTGTTTCA	60	50–55
LOC103986775	U-Box domain-containing protein 21-like	74	CTCATGTCGGCAACTCTCGT	CGTCCAAATCCCCAGACCTC	60	55–60
LOC103997576	Horcolin	78	TAACGCTGGGGACATCATCG	CCACCGTAGTGTTGGGTCTC	60	55–60
LOC103991351	Leucine-rich repeat extensin-like protein 4	81	CACCTGAGTGCATGGGGATT	CCCTAGAGCAAGCTACAGCC	60	55–60
LOC103971548	Cyclic dof factor 3-like	86	GCACAGGCTTCTGATCTGGT	GCCTTGACAAACCTTGCCTG	60	55
LOC103976748	CASP-like protein 4D1	89	AATGGCCTCTTCGACCAAGG	TGACGACGAGGGAAACCAAG	60	55
LOC103987539	U-Box domain-containing protein 21-like	89	CCACGCGATACGGAAGATGA	TCACCGGAATCTTAGGCGTG	60	55
LOC103990849	Serine/threonine-protein kinase	115	AACTGATCGGGTACTGCTGC	GACCACGGCAACGAAGAAAC	60	55
LOC103972371	Cycle dof factor 3-like	131	CGCACAGCTATTGCACGAAA	AGCTCCCAAATCACATGGCA	60	50
LOC103989093	Abscisic stress-ripening protein 2	139	TCGGAGACTGCCTACTCTGG	CGCCCATCTTTCCGAGATGT	60	55–60
LOC103985511	Trascription factor Bhlh 130	143	TCTCGTCGAGGCAAAACTCC	GGAATCGTCCCAAGAGCCAA	60	55
LOC103977424	Pectinesterase-like	150	GGGAACCACCGTTACGACAT	GCTGTTGGCAATGAGTGAGC	60	55

A large and diverse group of banana genes specifically triggered either by *P. fluorescens* Ps006 or by *B. amyloliquefaciens* Bs006 were genes associated with plant growth promotion, stress response and defense. Several of the genes identified in this study were connected to major plant growth stages and include: cytochrome C, U-Box domain-containing protein 21-like, and serine/threonine-protein kinase (embryonic development), leucine-rich repeat extensin-like protein 4, (cell differentiation and morphogenesis), CASP-like protein 4d1, transcription factor Basic Helix-Loop-Helix Protein bHLHP, pectinesterase-like (root growth), cytochrome C (nutrient transport), truncated transcription factor CAULIFLOWER and U-Box domain-containing protein 21-like (flowering), abscisic stress-ripening protein 2, as well as (fruit maturation) truncated transcription factor CAULIFLOWER and pectinesterase-like genes. Additionally, other genes were closely associated with plant growth promotion and include: cytochrome P450, caffeic acid 3-O-methyl transferase, horcolin, and cyclic dof factor.

### Genes related to plant promotion and growth

**Fig. 7:**
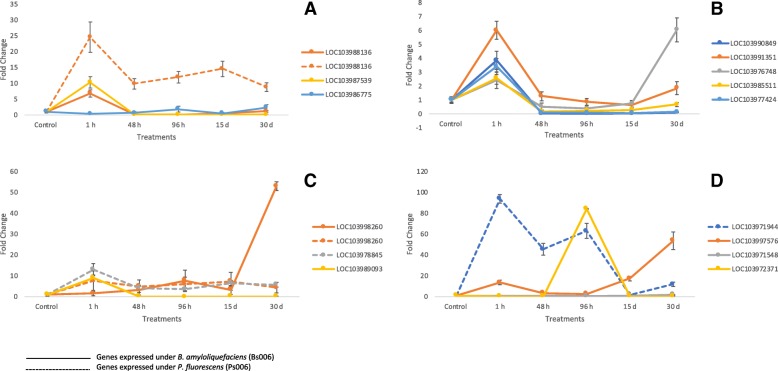
Genes expressed under *Bacillus amyloliquefaciens* (Bs006) and *Pseudomonas fluorescens* (Ps006) treatments. Genes related to growth promotion were expressed in ddPCR analyzes. (**a**) LOC103988136, LOC103987539, LOC103986775. (**b**) LOC103990849, LOC103991351, LOC103976748, LOC103985511, LOC103977424. (**c**) LOC103978845, LOC103989093, LOC103998260. (**d**) LOC103971944, LOC103997576, LOC103971548, LOC103972371

PGPR are involved in various biotic activities of the soil ecosystem to make it dynamic for turnover and sustainable for crop production [24]. They competitively colonize plant root systems and enhance plant growth through different mechanisms. Plant cytochrome C (CYTC) is preferentially expressed in root and apical meristems [25] and its knock-out results lethal and in an arrest of embryonic development. In the current in vitro study, with the presence of *B. amyloliquefaciens* Bs006 and *P. fluorescens* Ps006, seedlings of banana cv. Williams expressed the CYTC gene LOC103988136 that was transcriptionally overregulated 6.8-fold and 24.58-fold, respectively, at 1 h post-inoculation (Fig. 7a). Another activated gene found and belonging to the U-Box family was FB. This transcription factor belongs to one of the largest gene families related to cellular protein degradation (ubiquitinization), that regulate important processes such as cell growth, embryogenesis, floral development, and plant growth. In chickpea, more than 285 F-box genes are located in 8 chromosomes, showing a synteny with oat, *Lotus* and *Arabidopsis* genes [24]. The LOC103987539 gene was regulated 10.32-fold in the presence of *Bacillus amyloliquefaciens* Bs006 (Fig. 7a) and also the gene LOC103986775 elicited by *Bacillus amyloliquefaciens* Bs006 reported a fold change value of 2.34 (Fig. 7a). In this study we found that the gene LOC103990849 was overexpressed in the plants treated. This gene described as a serine/threonine-protein kinase, normally occurs early during somatic embryogenesis of banana growth. Recently, this gene has been observed as differentially expressed in banana, specifically in somatic embryogenesis routes [26]. Using a cDNA-AFLP approach, these researchers detected the early expression of kinase serine/threonine-protein kinase in growing cells in *Musa accuminata* ssp. *malaccensis* [26]. This gene showed a 3.84-fold expression difference in seedlings treated with *B, amyloliquefaciens* BS006 (Fig. 7b) in the banana cv. Williams transcriptome. Plant architecture is an essential component in its growth and development. There are several genes that target these structures, such as LRXI (LOC103991351), a leucine-rich repeat extensor gene that normally is expressed in radicular hair cells and that was up-regulated by PGPR in the banana transcriptome. It has also been suggested that LRX1 is an extracellular component of a mechanism that regulates root morphogenesis and elongation by controlling the polarized growth or the formation and assembly of the cell wall. In this study, LOC103991351 showed a 6.01-fold change in banana cv. Williams inoculated with *B. amyloliquefaciens* (Fig. 7b). The CASP-like protein gene 4D1 is a member of the Casparian Strip Membrane Domain Protein family and a transmembrane protein involved in plant growth [27]. CASP-like 4D1, LOC103976748 was transcriptionally regulated in the presence of Bs006, with a fold change value of 6.03 (Fig. 7b). CASP-like 4D1, as well as the extensin LRX3, are influenced by the PGPR presence and are both actively involved in the construction of the plant cell wall [27, 28]. Expression of the gene LOC103985511, corresponding to a transcription basic helix-loop-helix protein was overregulated 2.56-fold in plants treated with *B. amyloliquefaciens* Bs006 (Fig. 7b). This gene is known for its role in the maturation and growth of root hairs and trichomes [29]. The members of this family interact with inhibitory proteins (GL1, MYB23, WER) or activators (GLABRA 1) of the MYB family and factor WD40, such as the MYB-bHLH-WD40 complex, which regulates the appearance and growth of trichomes and root hairs. In banana, some of these transcription factors (TF), including MADS, bHLH, WRKY, AP2-EREBP, MYB-related and NAC domain TF families are highly expressed in ripe fruits [30].

Pectinesterase (PE) is a ubiquitous cell wall-associated enzyme thought to be responsible for the demethylation of galacturonyl residues in high-Mr pectin. This enzyme has been reported in many plant tissues and has been implicated in many developmental processes, including cellular adhesion, stem elongation [31], pollen tube development [32], abscission [33], and fruit ripening [34]. A differentially expressed pectinestarase gene LOC103977424 found in this transcriptome analysis, was overregulated in about 3.40-fold in seedlings treated with *B. amyloliquefaciens* Bs006 (Fig. 7b). Up-regulation of the truncated transcription factor CAULIFLOWER (CAL) gene LOC103978845 was found in the presence of *P. fluorescens* Ps006. CAULIFLOWER is a member of the factors that regulate flowering. In addition, *P. fluorescens* Ps006 regulated LOC103978845 by 12.93-fold (Fig. 7c). CAL in cauliflower is known as AP1, and its counterpart in *Arabidopsis thaliana* are two duplicate genes in plants of the *Brassicaceae* family. CAL and AP1 are factors of the MADS-box family that are involved in plant development, mainly in sepal and petal differentiation. CAL is expressed early in seed maturation and has an active expression at the time of floral meristem development [35]. In this study, the abscisic stress-ripening protein 2 (ASR) gene, LOC103989093, was expressed with an 8.96-fold change in banana cv. Williams seedlings treated with *Bacillus amyloliquefaciens* Bs006 (Fig. 7c). ASR plays a role in plants in the metabolism of lipids, carbohydrates, amino acids, energy, structural proteins, as well as in other functions. A study on *Musa acuminata* and *M. balbisiana* gene expression and protein analyses revealed that ASR gene expression would be detected in meristem cultures, roots, pseudostems and leaves [36]. Another gene found overregulated when plants were treated with *P. fluorescens* Ps006 and *B. amyloliquefaciens* Bs006 (a 7.63-fold and a 52.96-fold increase, respectively) was LOC103998260, a cyt P450 714B3 gene (Fig. 7c). This gene catalyzes extremely diverse reactions leading to the precursors of structural macromolecules such as lignin, cutin, suberin and sporopollenin. P450 has been reported as an important protein in the ripening stages of the *Cavendish* banana, where a protein accumulation occurs in the skin and pulp of mature fruits, but not in the roots, ovaries, flowers and leaves [37]. LOC103971944, a gene related to a caffeic acid 3-O-methyltransferase (COMT) was transcriptionally up-regulated (93.65-fold) in seedlings by effect of a treatment with *B. amyloliquefaciens* Bs006 (Fig. 7d). The COMT gene in angiosperms is involved in multistage methylation reactions of hydroxylated monomeric lignin precursors [38]. In plants as alfalfa, strong downregulation of COMT resulted in decreased lignin content, as well as in a reduction in the total guaiacyl (G) lignin units and a nearly total loss of syringyl (S) units in monomeric and dimeric lignin degradation products [39].

An increased transcription of a horcolin gene-like (LOC103997576) with 53.56-fold change was found in banana cv. Williams plants treated with *B. amyloliquefaciens* Bs006 (Fig. 7d). The product of this gene is a protein originally found in embryonic tissue of barley (*Hordeum vulgare*), a lectin with an affinity for mannose. Horcolin, as well as jacalin are actively induced by the presence of gibberellins [40], which has been reported to be induced in cucumber by the presence of the PGPRs *Burkholderia cepacia* SE4, *Promicomonospora* sp. SE188 and *Acinetobacter calcoaceticos* SE370. Similar to horcolin, the genes LOC103971548 and LOC103972371(Fig. 7d), annotated as Cyclic Dof factor 3, were found overexpressed in the current study. These genes were transcriptionally regulated in 1.73-fold and 84.4-fold, respectively, in plants stimulated by *B. amyloliquefaciens* Bs006. The Cyclic Dof factor 3 has been involved in embryonic development, seed germination, control of flowering [41, 42], branching of shoots, and development of pollen, fruit and vascular tissues [43–45]. Furthermore, Dof TFs also participate in carbon metabolism [46] and other physiological processes such as nitrogen assimilation, phytochrome and cytochrome signaling, as well as plant hormonal signaling. Additionally, this process is regulated by environmental factors such as light, temperature and nutrients, as well as by phytohormones, in particular gibberellins (GA) and abscisic acid (ABA). The corresponding gene of this protein has influence in Dof Affecting Germination protein (DAG1), a repressor of light-mediated seed germination processes [47]. The newly found Dof protein MaDof23 in banana functions as a transcriptional repressor, interacting also with the transcriptional activator MaERF29. Some authors have suggested that these two proteins control banana fruit ripening by working antagonistically to regulate 10 ripening-related genes involved in cell wall degradation and aroma formation [48].

The expression of all these genes described until now would suggest that both PGPR assessed, *P. fluorescens* Ps006 and *B. amyloliquefaciens* Bs006, trigger plant growth related genes at all levels, probably by increasing nutrient supply in the rhizosphere and/or stimulating the ion transport system in roots. Furthermore, also biological events such as electron transport, redox, lipid and carbohydrate metabolism. PGPR influence soil features and their transformation from a sterile and a low quality environment into an arable land, i.e. revitalizing soil quality and potentiating plant growth.

### Genes related to stress

Abiotic and biotic stresses are the most influential limiting factors for agricultural productivity. PGPRs are soil inhabitants that exhibit significant capabilities to mitigate abiotic and biotic stress [49]. In this study important genes involved in stress response linked to the presence of either *P. fluorescens* Ps006 or *B. amyloliquefaciens* Bs006 were identified. Among these, we find CYTC, CYT P450, AOX2, ACP, and HSPs, as well as the transcription factors MYB, WRKY, Basic Helix-Loop-Helix Protein bHLHP and U-Box domain-containing protein 21-like.

Cytochrome C, described above as one of the promoting genes that regulated growth in banana cv. Williams, is normally expressed in the roots of plant seedlings when they are subjected to osmotic stress. This gene is induced especially when the plant must meet the energy demand due to increased oxidative respiration and increased reactive oxygen species (ROS), or oxygen free radicals [50, 51]. The CYTC gene is one of the three genes known to be involved in banana plant mitochondrial respiration [52], and its function is essential in the early stages of plant development, probably because of its role in cyanide-sensitive respiration [25]. It is possible that these changes do not necessarily indicate a direct effect of the bacteria on the expression of CYTC, since the in vitro system in which the banana cv. Williams seedlings were grown exposed them to respiratory stress in the controlled atmospheric conditions used. P450 is known as other protein actively involved in attempting to detoxify oxygen free radicals actively released under the in vitro stress conditions, similar to those in which the banana cv. Williams seedlings were grown in the current study [37, 52].

**Fig. 8:**
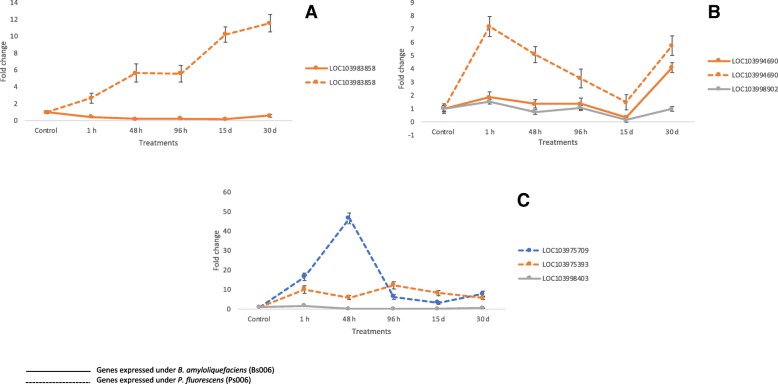
Genes expressed under *Bacillus amyloliquefaciens* (Bs006) and *Pseudomonas fluorescens* (Ps006) treatments. Genes related to stress were expressed in ddPCR analyzes. (**a**) LOC103983858. (**b**) LOC103994690, LOC103998902. (**c**) LOC103975709, LOC103975393, LOC103998403

Under stress conditions, as well as in senescence and hormonal response, plants trigger the expression of genes like the U-Box transcription factor. Another gene found differentially expressed in the current study was the Ubiquinol oxidase 2, mitochondrial-like gene (AOX2). The AOX2 gene corresponds to an alternative oxidase (Alternative Oxidase AO3). In our study, the gene LOC103983858 (AOX2) was influenced by the presence of *B. amyloliquefaciens* Bs006 and *P. fluorescens* Ps006, with 0.57-fold and 10.19-fold, respectively (Fig. 8a). AOX2, just like its AOX3 isoform, is a mitochondrial intermembrane protein that functions in the alternative respiration pathway that confers cyanide resistance and is an important redox balance sensor, allowing the plant to have activity under conditions of biotic and abiotic stress [53, 54]. However, AOX2 and AOX3 are not only stress response proteins, since they also have links to several metabolic processes that include carotenoid biosynthesis, chlorine respiration, electron transport in photosystem I (PSI), and works as a regulator in the photosystem II (PSII) [55]. As was described above, the ASR gene (LOC103989093) was expressed in seedlings treated with *Bacillus amyloliquefaciens* Bs006. In banana meristem cultures mAsr1 and mAsr3 genes have been found to be induced by osmotic stress and wounding, meanwhile mAsr3 and mAsr4 were expressed after plant exposure to ABA in banana.

Further, another gene regulated under stress conditions is the L-ascorbate oxidase homolog (AO). Mutations in this gene reduce the ability of the plant to respond to oxidative stress (reactive oxygen species) and to harmful levels of ozone [56, 57]. The AO enzyme is normally associated with glutathione and glutathione peroxidase activities. The associated AO-Glutathione, known as the heart of the redox hub, represents one of the major contributions to peroxide detoxification of hydrogen in the chloroplast [56, 57]. In the current study, the expression of the AO gene LOC103994690 resulted in a 4.09-fold change when banana seedlings were treated with *B. amyloliquefaciens* Bs006, meanwhile in the presence of *P. fluorescens* Ps006 it increased to 7.19-fold change (Fig. 8b). Additionally, another AO gene, LOC103998902, was also overexpressed in the presence of *B. amyloliquefaciens* Bs006 with 1.52-fold change value (Fig. 8b).

Additionally, the Heat shock cognate protein (HSPs) activity helps the correct formation of structures under thermal shock and protects against cellular function damage and during osmotic stress [58]. The production of HSPs), cold shock proteins and osmoregulation in bacteria regulates survival under adverse conditions. Furthermore, a small 70 KDa HSP was differentially expressed in the banana cv. Williams seedlings in the presence of *P. fluorescens* Ps006. The LOC103975393 gene showed a 12.5-fold change when overregulated in the presence of *P. fluorescens* Ps006 (Fig. 8c), meanwhile the LOC103975709 gene is up-regulated by 46.57-fold (Fig. 8C). In the case of plants responding to *B. amyloliquefaciens* Bs006, the gene LOC103998403 was up-regulated 1.57-fold (Fig. 8c).

### Genes related to defense

**Fig. 9:**
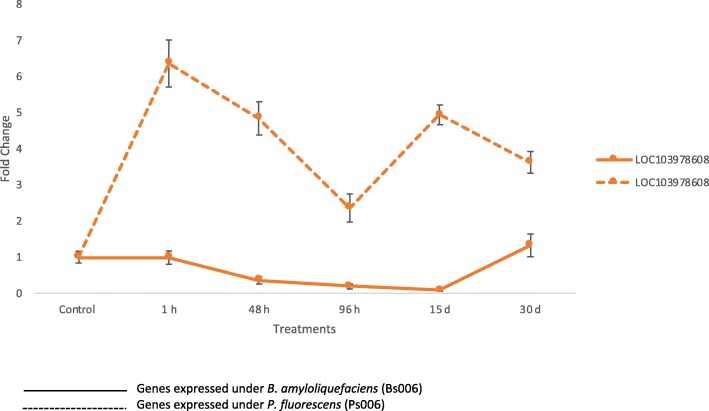
Genes expressed under *Bacillus amyloliquefaciens* (Bs006) and *Pseudomonas fluorescens* (Ps006) treatments. Genes related to defense were expressed in ddPCR analyzes. LOC103978608

Plants establish beneficial, harmful, or neutral relationships with bacteria. PGPRs may act as molecular cues influencing plant defense [59]. One of the characteristics of the defense response of plants is the production of genes related to a wound-induced protein 1-like: The wound gene LOC103978608 belongs to a family of plant wound-induced protein sequences related to WI12 from *Mesembryanthemum crystallinum*. WI12 preferentially accumulates in the cell wall and may play a role in the reinforcement of cell wall composition after wounding occurs and during plant development [60]. This gene has been reported to be expressed in banana by both PGPR used in this study, suggesting its potential in an eventual response of the plant to damage caused by wounds or attack by pathogens. Following the treatment with *B. amyloliquefaciens* BS006, a gene 1.32-fold-change occured, whereas with plants treated with *P. fluorescens* PS006, the change increased reaching a 6.35-fold (Fig. 9).

## Discussion

Plants have always been in a symbiotic relationship with soil microbes (bacteria and fungi) during their growth and development. The symbiotic microorganisms of the free-living soil that inhabit the rhizosphere of many plant species have various beneficial effects on the host [61]. PGPR influence plant growth through direct and indirect mechanisms that contribute to improve the resistance of the plant to different pathogens.

In the case of genes related to plant promotion and growth (maturation, flowering, elongation, structure, differentiation and development), it is known that several mechanisms such as nutrient absorption or increase of nutrient availability by nitrogen fixation, mineralization of organic compounds, solubilization of mineral nutrients and production of phytohormones participate in plant growth [62]. Variations in plant response may be related to the interacting microbial strains or even the plant species. Direct mineral absorption enrichment occurs due to increases in individual ion fluxes on the root surface in the presence of PGPR, thus, increasing nutrient availability. These statements are supported by the fact that previous to this study, ca. 14 genes involved in the synthesis of cofactors, 25 genes implicated in potassium metabolism, 52 genes involved in nitrogen metabolism and 78 genes associated to phosphorus metabolism have been reported in the genome of *P. fluorescens* Ps006. In the case of *B. amyloliquefaciens* BS006, 20 genes are involved in the synthesis of cofactors, 51 in vitamins, 20 in pigments, 9 are involved in the metabolism of potassium, 31 in nitrogen metabolism and finally, 31 are involved in phosphorus metabolism [21, 22].

The expression of stress and defense genes in plants would be mediated by the action of PGPR, involving processes that helps plants to actively grow under abiotic or biotic stress conditions [63]. PGPR induce the production of repressive substances that increase the natural resistance to pathogens and pests [64]. These microorganisms participate in plant defense by producing hydrolytic enzymes as chitinases, cellulases and proteases, as well as various antibiotics, volatile organic compounds (VOC), exopolysaccharides (EPS) and siderophores that protect plants against pathogens, among others [24, 65].

The strain *P. fluorescens* Ps006 used in the current study in banana, has a genome that contains 193 genes for the response to environmental stress, as well as genes involved in plant health and defense, described as follows: 14 genes for bacteriocins and antibacterials synthesized ribosomally (peptides), 123 genes for resistance to antibiotics and toxic compounds, and 12 genes for invasion and intracellular resistance. On the other hand, the PGPR genome of *B. amyloliquefaciens* Bs006 contains genes related to plant stress, health and defense such as: 1 adhesion, 16 bacteriocins and antibacterial peptides synthesized ribosomally, 37 genes for antibiotic resistance and toxic compounds, 17 genes that regulate invasion and intracellular resistance, and 117 genes involved in latency and sporulation.

Based on the aforementioned, all these PGPR genes would have effects in the banana transcriptome outcome, since they are known to generate and elicit a specific response in plants. To better understand the direct and indirect interactions between banana roots and rhizobacteria, this study defined global transcriptome profiles at 0, 48 and 96 h after inoculation with the bacterial strains. The treated banana plants were compared with the control (plants not inoculated) at 0 h after bacterial exposure, and genes were considered differentially expressed when the FDR was < 0.05. The response of banana cv. Williams to *P. fluorescens* showed an increasing number of DEGs over time, i.e. 1027, 1398 and 3541 at 0, 48 and 96 h, respectively. Conversely, plants inoculated with *B. amyloliquefaciens* showed a decrease in the number of DEGs over time: 2652, 1530 and 1250 at 0, 48 and 96 h, respectively. These differences suggest that *Pseudomonas* and *Bacillus* colonize differently banana plant roots. Correlating these data with the colonization pattern evaluated through scanning electron microscopy (SEM) and confocal microscopy, the colonization took place preferably in defined regions of the elongation and differentiation zones of plant roots, as well as in lateral roots and the unions between roots. These results are consistent with those of a previous report published by Liu et al., 2016. In this study, the cells of *B. amylolyquefaciens* Bs006 colonize externally and begins with a very copious growth but decreases in the final evaluation times. Bacteria obtain several advantages when living in biofilms, including protection against predation, drying and exposure to antibacterial substances, and better acquisition of nutrients present in the plant environment [66]. According to authors biofilms provide survival sites for beneficial and opportunistic bacteria, also providing protection and increasing the potential of bacteria to survive and evolve in the plant environment [68]. Biofilms have been shown to improve (i) the behavior of individual bacteria or several members in the soil microbial community, and (ii) the health and productivity of plants as a result of the whole microbiome-host interaction [67]. This group suggests that somehow, the interaction and exchange of nutrients in the case of *B. amyloliquefaciens* Bs006 (external to the root) are depleted and show temporality. Moreover, in our study, bacterial cellulose could not be found within the banana root tissue, which indicates that the *Bacillus* strain used was not a banana root endophyte. In disease control studies, the population of the biological control agent decreased 15 days after inoculation, which was consistent with a previous study published [68]. Perhaps this reduction in the apparent population of the biological control agent resulted from the competition of indigenous microorganisms in the soil or the loss of plasmids [68].

This study corroborated that the colonization pattern of *P. fluorescens* Ps006 was endophytic, namely, that it was able to colonize inside the roots. This strain showed few signs and colonization in the roots during the first hour and after 48 h; however, and in contrast, after 96 h the bacterium initiated the endophytic colonization of the root. Somehow, this bacterium manages to enter into the plant and uses the entire energy, enzymatic and nutrient battery in a more stable way by being inside the plant. It is known that after penetration, the rhizobacteria/endophytes can move towards the xylem and be transported systemically to stems and leaves [69]. The surface of the bacterial cell would play a key role in bacterial aggregation, which in turn promotes bacterial dispersion, survival, and the ability to adhere to plant surfaces. Without any apparent damage to the plant, the endophytic colonization caused by bacteria would reflect their ability to adapt selectively to specific ecological niches that favor an intimate microbe-host association [70, 71]; furthermore, by using confocal laser scanning microscopy (CLSM) it was possible to demonstrate in olive root that cells of *Pseudomonas* spp. actively moved within the hair of the root quickly multiplying inside and progressing along the hair. In vines it has been reported that strain *Pseudomonas* PsJN colonized the rhizosphere, penetrated the roots and then migrated through all plant tissues within 94 h of bacterial application [70]. These reports are similar to the findings of this study, where the bacterial colonization of banana roots by *Pseudomonas* Ps006, became endophytic 96 h after inoculation. The similar colonization patterns of Ps006 observed in the current experiments would indicates that some in vitro microbe-host models could approach real soil conditions to a certain extent.

Additionally, in the validation of the genes by ddPCR the same DEGs behavior was also evidenced. A very high initial expression of *B. amyloliquefaciens* induced banana genes that was not persist in time because it decreased, compared to the response of *P. fluorescens*, which showed a timid initial response that was potentiated in the intermediate and final evaluation times. The tendency in many banana genes expressed by *P. fluorescens* interaction was maintained with a high value change in expression patterns, and most interestingly, persisted in time. The differences in banana gene expression induced by the two PGPR could be explained by contrasting colonization models showed by the two microorganisms.

With this study, the first banana cv. Williams transcriptome elicited by the PGPRs *B. amyloliquefaciens* Bs006 and *P. fluorescens* Ps006 is presented, contributing to a better understanding of the biological process that participates in these microbe-plant interactions.

## Conclusions

Bananas are an economically important fruit in many parts of the world. In the current study, the transcriptome of banana cv. Williams was sequenced in response to *Bacillus amyloliquefaciens* strain Bs006 and *Pseudomonas fluorescens* strain Ps006. Twenty-two genes were validated experimentally using ddPCR; these genes were categorized into several types of functions associated with promotion and growth, stress and defense.

In recently published data carried out by our laboratory, colonization studies conducted in banana with *B. amyloliquefaciens* and *P. fluorescens* revealed that both bacteria behave differently when colonizing plant roots [72]. *B. amyloliquefaciens* showed colonization on the surface of the roots through biofilms; whereas *P. fluorescens* showed an alternative colonization pattern extending as an endophyte that grows inside the roots.

Although a high colonization rate was found at the early stages of the interaction between *B. amyloliquefaciens* and the plant, it decreased over time. In contrast, the abundance of *P. fluorescens* increased gradually after inoculation. These observations suggest that somehow the interaction and exchange of nutrients in the case of *B. amyloliquefaciens* Bs006 (external to the root) decrease, showing temporality. *P. fluorescens* Ps006 conversely, colonizes endophytically banana roots 96 h after inoculation. This would reflect their ability to adapt selectively to specific ecological niches that favor a more intimate and stable association over time between *P. fluorescens* Ps006 and banana. Banana plants inoculated with *P. fluorescens* showed an increasing number of differentially expressed genes (DEGs) over time: 1027, 1398 and 3541 at 0, 48 and 96 h, respectively. On the other hand, the banana response to *B. amyloliquefaciens* showed a decrease in the number of DEGs over time: 2652, 1530 and 1250 at 0, 48 and 96 h, respectively (see Fig. 2). These differences suggest that *Bacillus* and *Pseudomonas* trigger a contrasting response that is closely related to their root colonization patterns in banana. The same trend was evidenced in the validation of the genes by ddPCR evaluated 15 and 30 days after inoculation.

## Methods

### Plant material

Banana (*Musa acuminata* Colla) cv. Williams (Cavendish subgroup, genome AAA) was used in this study. Mother plants of the Williams cultivar were obtained from the ex vitro Musa Bank at the research center C.I. Caribia, AGROSAVIA. Murashige and Skoog (MS) culture medium [73] supplemented with 0.1 g/l of myoinositol, 3 ppm of benzyl amino purine (BAP), 0.5 ppm of indole acetic acid (IAA), 1 ppm of vitamins (thiamine hydrochloride), and 30 g/l of sucrose was used, and adjusted to pH 5.7, for the micropropagation of banana seedlings (meristem extraction) in the laboratory. Seedlings were placed in the growth room under controlled conditions, with a temperature of 23 °C ± 1 °C during the day and 20 °C ± 1 °C during the night, at 16/8-h light/dark photoperiod, and with a relative humidity of 65% ± 10% for four weeks. The Murashige and Skoog (MS) culture medium was also used for the in vitro propagation and rooting of seedlings.

### Bacterial strains

*Bacillus amyloliquefaciens* strain Bs006 [21] and *Pseudomonas fluorescens* strain Ps006 [22] were isolated from *Physalis peruviana* roots and from *Furcraea* spp. in the departments of Boyacá and Cauca, Colombia, respectively. These two rhizobacteria were evaluated at the greenhouse level in banana cv. Williams plants for growth promotion. The bacterial strains were cultured on Luria Bertani agar medium (LBA; Sigma Chemical Company, St. Louis, Missouri, USA) and stored in LB liquid medium (LB; Sigma) with 15% glycerol at − 80 °C. The final inocula were obtained from a single colony inoculated in LB medium cultured in constant shaking at 28 °C for 48 h, and then adjusted to a concentration of 10^8^ CFUs ml^− 1^ with sterile distilled water.

### Planting and microbial inoculation

Seedlings of around 3 cm of height with three fully expanded leaves were transplanted into trays of 24 cones containing a substrate mixture with a proportion of 3: 1 (sand: alluvion). Substrate mixture was sterilized with Agrodine® (fungicidal and bactericidal) using 3 ml L^− 1^ of water for each 18 kg of substrate. Seedlings developed in the growth room under controlled conditions with a temperature of 23 °C/20 °C ± 1 °C day/night, with a 16/8-h light/dark photoperiod, and with a relative humidity of 65% ± 10% for a maximum period of 30 days (to perform the samplings in the five sampling times established). In vitro plants were inoculated once with 5 ml (10^8^ CFUs ml^− 1^) of the *B. amyloliquefaciens* strain Bs006 and *P. fluorescens* strain Ps006 inoculants. For experimental controls, 5 ml of sterile distilled-deionized water was added to plant preparations. For the purposes of sequencing the transcriptome in this investigation, three sampling times were chosen for three biological replicates at 1, 48 and 96 h. However, considering the growth time in the greenhouse (up to 30 days), gene expression was experimentally validated at five sampling times: 1, 48, and 96 h, and additionally after 15 and 30 days (Additional file 5, Figure S5).

### Experimental design and sampling

For the transcriptomic experiments, whole seedlings with three biological replicates per treatment were inoculated with two rhizobacteria strains (*B. amyloliquefaciens* strain Bs006 and *P. fluorescens* strain Ps006) at 1, 48, and 96 h, and after 15 and 30 days. Each treatment was removed from the container and macerated in liquid nitrogen for processing.

### RNA extraction

For each treatment, three biological replicates were used (three banana plants). A total of 45 corresponding samples were macerated in liquid nitrogen and transferred to 2 ml sterile microfuge tubes. Cold (4 °C) Plant RNA Reagent (600 μl; Invitrogen, Carlsbad, San Diego, USA) was added to the tubes followed by vortexing for 1 min and were then incubated on an inclined mixer for 5 min at room temperature. Samples were centrifuged at 12,000 g for 3 min at room temperature following the procedure according to Yockteng et al. [74]. The supernatant was transferred to a Phase Lock Gel tube (5 Prime, Gaithersburg, Maryland, USA) or a sterile RNase-free (USA Scientific, Inc., Ocala, Florida, USA) 2 ml microfuge tube. One hundred μl (100 μl) of sterile 5 M NaCl and 300 μl of chloroform-isoamyl alcohol (24:1) were added to each tube, and were well mixed by inversion; then, these were centrifuged at 12,000 g for 10 min at 4 °C. The aqueous (upper) phase was transferred to a sterile RNase-free microfuge. Four hundred and fifty microliters (450 μl) of LiCl 4 M (Fisher Chemical, New Jersey, USA) and 150 μl of isopropyl alcohol were added, and the tube was inverted, allowing total RNA to precipitate at − 20 °C for 1 h. Tubes were then centrifuged at 12,000 g for 25 min at 4 °C. Supernatants were removed with micropipettes being careful not to disturb the pellets. One milliliter (1 ml) of 75% (v/v) cold ethanol was added to the pellets followed by centrifugation at 12,000 g for 5 min at 4 °C. The ethanol was withdrawn with micropipette and the tubes were centrifuged again for 2 min to collect the residual liquid. Tubes were allowed to dry on ice for 15 min and the pellets were re-suspended in 20 μl of RNase-free water. To verify the quality of the RNA an electrophoresis gel was run and the degree of integrity (RNA integrity number RIN) of 7 or more was measured [74] using the Agilent 2100 Bioanalyzer (Agilent Technologies, Inc., Waldbronn, Germany).

### *Musa acuminata* transcriptome sequencing

For the development of genomic libraries, the SureSelect Strand-Specific RNA Library Prep Kit for Illumina Multiplexed Sequencing was used (Agilent Technologies, Santa Clara, California, USA), following the manufacturer’s instructions. To establish the quality of the amplified product, the Agilent 2100 Bioanalyzer (Agilent Technologies, Waldbronn, Germany) and Agilent DNA 1000 kit (Agilent Technologies) were used. The concentration of the library under a range of 200 to 400 bp peaks was monitored. The library construction was done with 200 bp inserts, 100 bp paired-end sequencing and 40 M reads per sample. Sequencing of the *Musa acuminata* transcript was performed in the Illumina Hi Scan SQ™ (Towne Center Drive, San Diego, California, USA).

### Preprocessing and quality control of RNA-seq

The Trimmomatic program version 0.32 [75] was used to remove sequence adapters and low quality bases using a sliding window length 4, cutting when the average Phred quality score fell below 20. The FastQC software version 0.11.2 (https://www.bioinformatics.babraham.ac.uk/projects/fastqc/) was used for quality control, before and after trimming.

### Differential gene expression

The Banana Reference Sequence (RefSeq) genome and annotation files were retrieved from the National Center for Biotechnology Information (NCBI) included in the Genomes FTP repository (ftp://ftp.ncbi.nlm.nih.gov/genomes/Musa_acuminata/). Paired-end reads were mapped against the banana genome using the HISAT2 program version 2.0.0-beta [76] (https://ccb.jhu.edu/software/hisat2/index.shtml) with the *--no-unal* option and other parameters as default. Reads mapped to each gene feature were counted using the HTSeq software version 0.6.0 [77] with default parameters. Raw count tables from 27 samples were merged and low-expressed genes (genes with less than 1 read per million of reads in at least 3 samples) were removed prior to statistical analysis. Raw count data were normalized using the trimmed mean of M values (TMM) method [78]. Differential expression analyses were carried out using edgeR software version 3.10.5 [78]. Pairwise comparisons of three biological replicates belonging to each group were made compared to the 1-h control group as reference. *P*-values were adjusted using the Benjamini-Hochberg procedure [78] and genes with a false discovery rate (FDR) < 0.05 were considered differentially expressed genes (DEGs).

### Gene ontology (GO) annotation and enrichment

Banana protein-coding genes were mapped with the Gene Ontology Plant Slim-terms using the Blast2GO software version 3.0.11 [79]. Gene Ontology (GO) categories enriched in the lists of DEGs of banana were identified with the goseq software version 1.20.0 [80] and using the list of expressed genes in all samples as background. P-values provided by goseq were adjusted for multiple testing using the Benjamini-Hochberg procedure, and genes with a false discovery rate (FDR) < 0.05 were considered GO-enriched terms.

### Primer design for gene validation using droplet digital PCR (ddPCR)

Primers were designed using Primer-BLAST at NCBI (https://www.ncbi.nlm.nih.gov/tools/primer-blast/). The design parameters employed are as follows: a) Product size = 70–150 bp; b) Tm = 58 – 62 °C with a maximum Tm difference of 2 °C; c) Search of pairs of primers specific to the desired PCR template was enabled. In this case ddPCR, d) Database = RefSeq mRNA Organism *Musa acuminata subsp. malaccensis* (taxid: 214687).

### Gene validation using droplet digital PCR (ddPCR)

#### Synthesis of cDNA

Five hundred nanograms (500 ng) of total RNA was used with reverse transcriptase in a 20-μl reaction mixture using SuperScript™ reverse transcriptase (Invitrogen) and 50 μM oligo dT, according to the manufacturer’s instructions. The reaction was incubated at 55 °C for 10 min, then inactivated at 80 °C for 10 min. To remove the RNA, 1 μl of *E. coli* RNase H was added and followed by incubation at 37 °C for 20 min. Product was quantified with the NanoDrop™ 2000 (Thermo Scientific, Wilmington, Delaware, USA), obtaining concentrations between 567 and 1222 ng/μl. Samples were stored at − 20 °C until used.

#### Droplet digital PCR (ddPCR)

*Musa acuminata* cDNA was quantified using the QX200™ Droplet Digital™ PCR system (Bio-Rad, Pleasanton, California, USA). The reaction mixture for Digital Droplet PCR (ddPCR) is as follows: 12 μl of ddGCR™ EvaGreen Supermix; 1.2 μl of the forward primer (250 nM); 1.2 μl of the reverse primer (250 nM); 4.6 μl of molecular grade water; and 5 μl of cDNA, for a final volume of 24 μl. The mixture was placed in an 8-channel DG8™ Droplet Generator Cartridge (Bio-Rad Laboratories. Hercules, California, USA) and 70 μl of droplet generation oil was added to the QX200™ Droplet Generator. Samples and oil were combined in the microchannels of the cartridge to generate an emulsion of up to 20,000 drops per sample. The oil droplet suspension was transferred to a 96-well microtiter plate and placed in the thermocycler. Cycle conditions were as follows: 95 °C for 5 min, 40 cycles of 95 °C for 30 s, and 59 °C for 1 min. Then, a stabilization phase was applied at 4 °C for 5 min followed by an increase to 90 °C for 5 min. The drops were then read automatically by the QX200 Droplet Reader using the QuantaSoft™ software (Bio-Rad).

## Additional Files

Additional File 1: **Figure S1.** Quality control analysis. Total number of millions of reads after trimming adapters and low-quality bases (upper panel). Percentage of mapped reads to the banana genome (bottom panel). Samples inoculated with *B. amyloliquefaciens* (Bs006), *P. fluorescens* (Ps006), and the control (no PGPR inoculated) are depicted in blue, red, and green, respectively. (PDF 24 kb)

Additional File 2: **Table S2.** Statistics and list of differentially expressed genes in samples inoculated with *B. amyloliquefaciens* (Bs006). Results at different analysis times (1 h, 48 h, 96 h), shared genes between times, and those exclusively expressed at a specific time are in different tabs. (XLS 10271 kb)

Additional File 3: **Table S3.** Statistics and list of differentially expressed genes in samples inoculated with *P. fluorescens* (Ps006). Results at different analysis times (1 h, 48 h, 96 h), shared genes between times, and those exclusively expressed at a specific time are in different tabs. (XLS 10493 kb)

Additional File 4: **Table S4.** Statistics and list of differentially expressed genes shared between *B. amyloliquefaciens* (Bs006) and *P. fluorescens* (Ps006). Results at different analysis times (1 h, 48 h, and 96 h) are in different tabs. (XLS 2503 kb)

Additional File 5: **Figure S5.** (A) Clusters of banana seedlings inside the container. (B) Clusters of banana seedlings outside the container. (C) Individualization and rhizobacteria inoculation processes. (D) Plants inoculated with *Bacillus*. (E) Plants inoculated with *Pseudomonas*. (F) Control plants. Maintained in controlled conditions of temperature and photoperiod and sampled according to defined times: 1, 48, and 96 h, and 15 and 30 days after inoculation. (PDF 10883 kb)

## Abbreviations

ddPCR: droplet digital PCR
DEG: Differentially expressed genes
PGPR: Plant growth-promoting rhizobacteria
