# Supplementary figures and images for: Banana (Musa acuminata) transcriptome profiling in response to rhizobacteria: Bacillus amyloliquefaciens Bs006 and Pseudomonas fluorescens Ps006
Source: BMC Genomics. 2019 May 14;20:378. doi: 10.1186/s12864-019-5763-5 (PMC6518610; doi:10.1186/s12864-019-5763-5)

**Total Number of Reads**

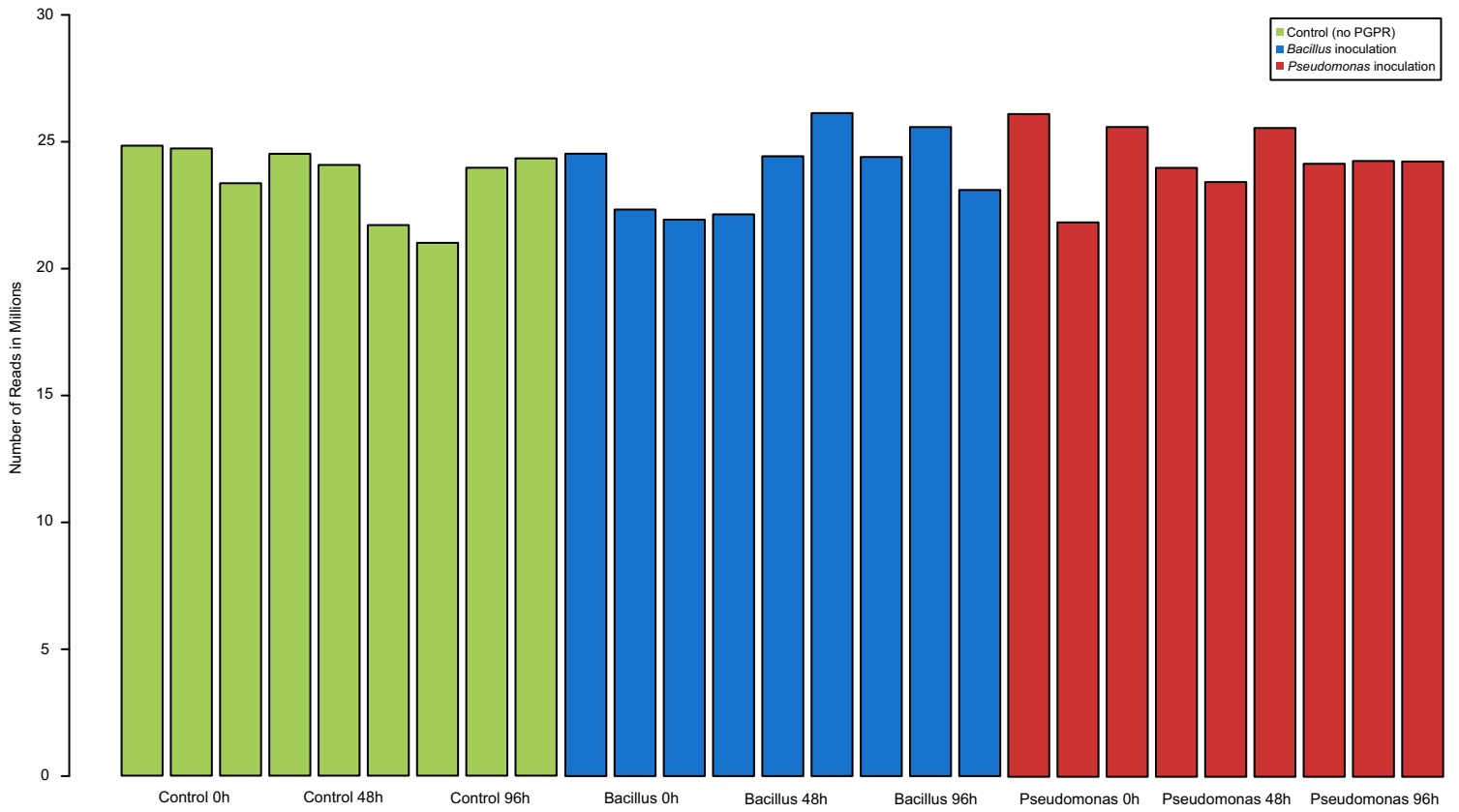

**Percentage of Mapped Reads**

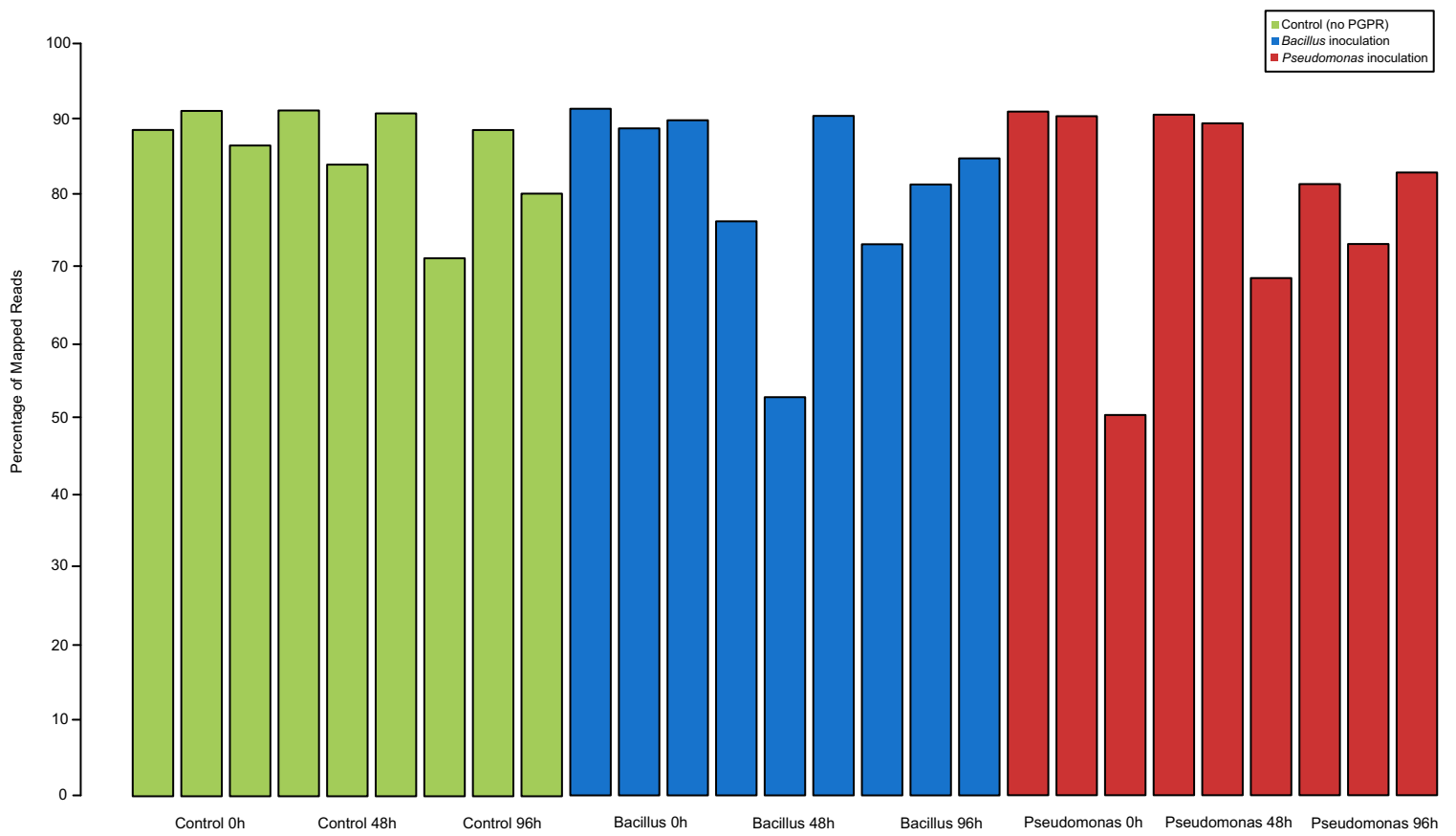

Supplement: Supplementary file 1 — Figure S1. Quality control analysis. Total number of millions of reads after trimming adapters and low-quality bases (upper panel). Percentage of mapped reads to the banana genome (bottom panel). Samples inoculated with B. amyloliquefaciens (Bs006), P. fluorescens (Ps006), and the control (no PGPR inoculated) are depicted in blue, red, and green, respectively. (PDF 24 kb) [file 12864_2019_5763_MOESM1_ESM.pdf]

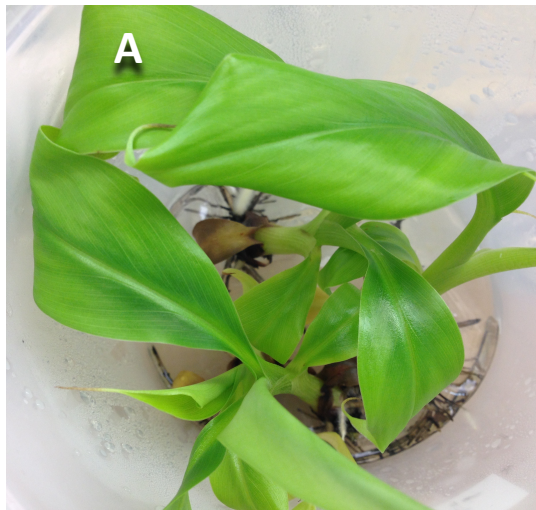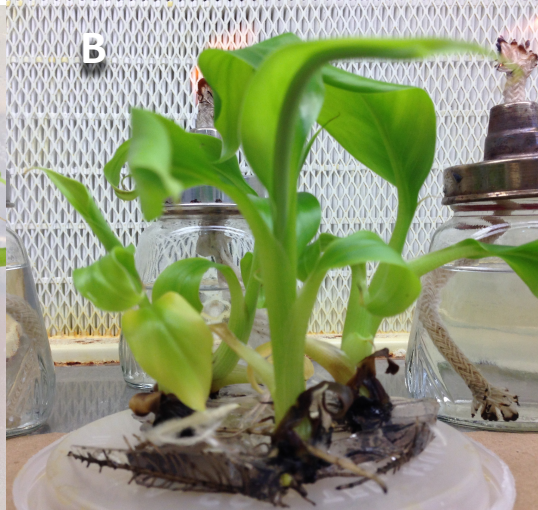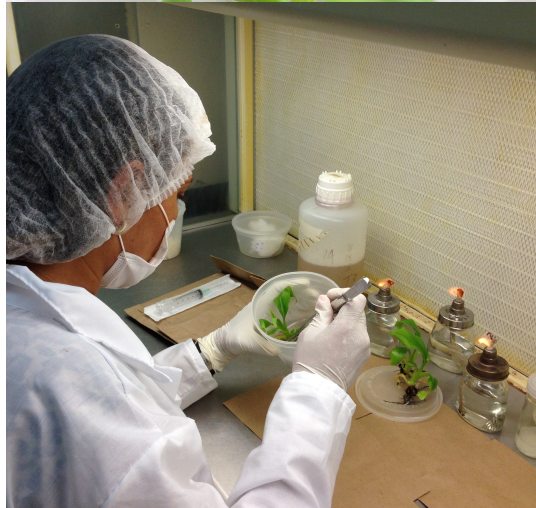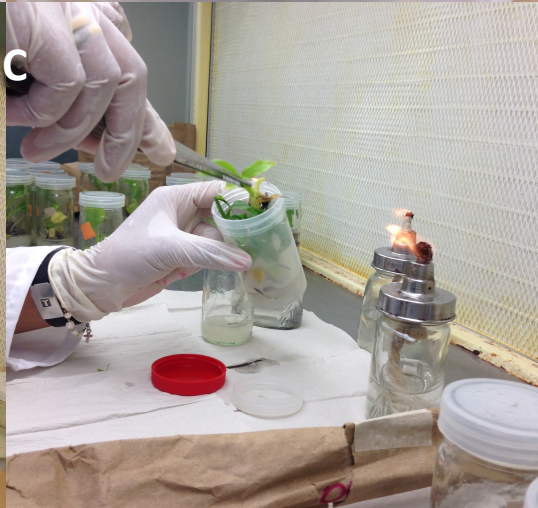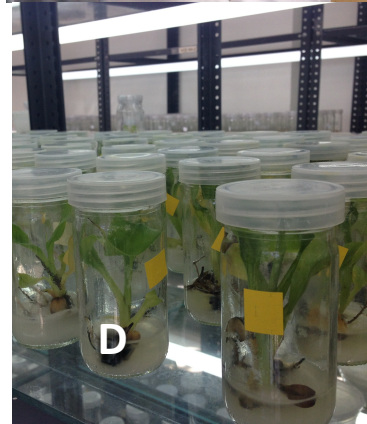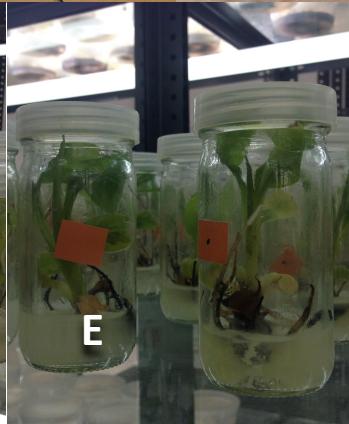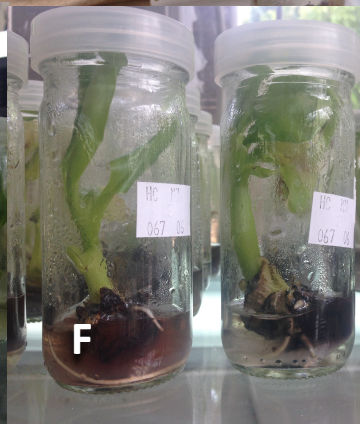

Supplement: Supplementary file 5 — Figure S5. (A) Clusters of banana seedlings inside the container. (B) Clusters of banana seedlings outside the container. (C) Individualization and rhizobacteria inoculation processes. (D) Plants inoculated with Bacillus. (E) Plants inoculated with Pseudomonas. (F) Control plants. Maintained in controlled conditions of temperature and photoperiod and sampled according to defined times: 1, 48, and 96 h, and 15 and 30 days after inoculation. (PDF 10883 kb) [file 12864_2019_5763_MOESM5_ESM.pdf]
